# Analyzing Empowerment Processes Among Cancer Patients in an Online Community: A Text Mining Approach

**DOI:** 10.2196/cancer.9887

**Published:** 2019-04-17

**Authors:** Suzan Verberne, Anika Batenburg, Remco Sanders, Mies van Eenbergen, Enny Das, Mattijs S Lambooij

**Affiliations:** 1 Leiden Institute of Advanced Computer Science Leiden University Leiden Netherlands; 2 Centre for Language Studies Radboud University Nijmegen Netherlands; 3 Amsterdam School of Communication Research University of Amsterdam Amsterdam Netherlands; 4 Department of Research Netherlands Comprehensive Cancer Organization Utrecht Netherlands; 5 Department of Health Services Research and Health Economics National Institute of Public Health and the Environment Bilthoven Netherlands

**Keywords:** cancer, health communication, online social networking, empowerment, text mining, machine learning

## Abstract

**Background:**

Peer-to-peer online support groups and the discussion forums in these groups can help patients by providing opportunities for increasing their empowerment. Most previous research on online empowerment and online social support uses qualitative methods or questionnaires to gain insight into the dynamics of online empowerment processes.

**Objective:**

The overall goal of this study was to analyze the presence of the empowerment processes in the online peer-to-peer communication of people affected by cancer, using text mining techniques. Use of these relatively new methods enables us to study social processes such as empowerment on a large scale and with unsolicited data.

**Methods:**

The sample consisted of 5534 messages in 1708 threads, written by 2071 users of a forum for cancer patients and their relatives. We labeled the posts in our sample with 2 types of labels: labels referring to empowerment processes and labels denoting psychological processes. The latter were identified using the Linguistic Inquiry and Word Count (LIWC) method. Both groups of labels were automatically assigned to posts. Automatic labeling of the empowerment processes was done by text classifiers trained on a manually labeled subsample. For the automatic labeling of the LIWC categories, we used the Dutch version of the LIWC consisting of a total of 66 word categories that are assigned to text based on occurrences of words in the text. After the automatic labeling with both types of labels, we investigated (1) the relationship between empowerment processes and the intensity of online participation, (2) the relationship between empowerment processes and the LIWC categories, and (3) the differences between patients with different types of cancer.

**Results:**

The precision of the automatic labeling was 85.6%, which we considered to be sufficient for automatically labeling the complete corpus and doing further analyses on the labeled data. Overall, 62.94% (3482/5532) of the messages contained a narrative, 23.83% (1318/5532) a question, and 27.49% (1521/5532) informational support. Emotional support and references to external sources were less frequent. Users with more posts more often referred to an external source and more often provided informational support and emotional support (Kendall τ>0.2; *P*<.001) and less often shared narratives (Kendall τ=−0.297; *P*<.001). A number of LIWC categories are significant predictors for the empowerment processes: words expressing assent (*ok* and *yes*) and emotional processes (expressions of feelings) are significant positive predictors for emotional support (*P*=.002). The differences between patients with different types of cancer are small.

**Conclusions:**

Empowerment processes are associated with the intensity of online use. The relationship between linguistic analyses and empowerment processes indicates that empowerment processes can be identified from the occurrences of specific linguistic cues denoting psychological processes.

## Introduction

### Background

Peer-to-peer online support groups and the discussion forums in these groups can help patients by providing opportunities for improving their empowerment [1-4]. We adopt our definition of empowerment from the work by Van Uden-Kraan et al [1,5]. Empowerment is a process by which patients gain mastery over their situation [1,5-7]. Previous studies found that peer-to-peer online platforms can be sources of information and emotional support, both being empowerment processes [8-13]. Online empowerment processes can facilitate empowerment outcomes outside the online environment such as being better informed, feeling more confident with the physician, and improved acceptance of the disease [1].

Most previous research on online empowerment and online social support uses qualitative methods or traditional questionnaires and interviews to gain insight into the complex dynamics of online empowerment processes [1,4,5,10,12,14]. These studies provide knowledge on empowerment processes, underlying mechanisms, and empowerment outcomes. In addition to these qualitative methods, it is valuable to systematically investigate the written communication between patients using automated text analysis methods. Automated analysis allows (1) more consistent and reproducible coding of user-generated content and (2) the scaling of the analysis to larger corpus sizes. This helps the research community to gain knowledge about general patterns and possible differences within and between patient communities. If this void is filled, it will generate knowledge about the presence of empowerment processes in online patient communities, the relation to online patient activities, and the differences between groups of patients. An important feature of this type of research is the use of unsolicited data, enabling to study natural use of language in patient communities. Within patient communities, we focus on the discussion forums (hereafter called *forums*) of people affected by cancer.

We use the qualitative work of Van Uden-Kraan et al [1,5] on the empowerment of users of online patient support groups as the basis of our study. According to these authors, there is an important difference between empowerment *processes* and empowerment *outcomes*. Empowerment processes are processes that occur on the online forum itself, manifesting as the online communication between patients (eg, as exchanging information and sharing experiences). Examples of *empowerment processes* occurring within the online environment are exchanging information, encountering emotional support, finding recognition, sharing experiences, helping others, and amusement. Empowerment outcomes occur mostly *outside the online environment*, that is, these processes help patients to feel better informed or feel more confident about their treatment (examples of empowerment outcomes). Examples of *empowerment outcomes* mentioned by patients are being better informed; feeling confident with their physician (better shared decision making), their treatment, and their social environment; improved acceptance of the illness; increased optimism and control; and enhanced self-esteem, social well-being, and collective action. As our goal was to distill the concept of empowerment from the data that are available in online discussion forums, we focus on the *empowerment processes* in this study.

### Prior Work

#### Defining Empowerment in Patient Support Groups

In this study, the messages posted in discussion forums were categorized based on empowerment processes defined in previous work [5,15-17]. The processes that we distinguish are listed and explained below:

1. Narrative: Patients share their disease and treatment history with their fellow users [15], often including emotions. Sometimes, they contain a reflection of one’s life after the disease or have religious or spiritual references [16,17]. This empowerment process is included in this study as *narrative*.
2. Question asking: Users might ask questions (requests for information or support) to the community, to reach out for advice [5]. This empowerment process is included in our study as *question.*
3. Providing information: Informational support is provided if one shares *factual* information learned from their own experiences to help others (eg, information about cancer, the prognosis, or insurances [5]). This empowerment process is included in our study as *informational support*.
4. Providing emotional support (including esteem support, network support, affective support, and tangible support): Users can emotionally support each other, recognize and understand each other’s feelings, and by doing that help one another [5,15,17]. This empowerment process is included in our study as *emotional support.*
5. Reference to external source of information: Due to the nature of the internet as an interlinked network, users can refer patients to external sources of information [16,17]. For instance, questions about how health care insurance works when receiving treatment can be answered by referring to information on an insurance company’s website. This empowerment process is included in our study as *external source.*

#### Automated Text Analysis in Empowerment Studies

Previous text mining studies show that it is possible to identify (disease-related) topics that are discussed online. In particular, Wang et al [18] used text mining techniques to quantitatively analyze online activity related to empowerment. They found that people use online communities mainly to share their personal story and subsequently become less active in the community, in terms of posting messages. Chou et al found that over time (2003, 2005, and 2008), the percentage of cancer survivors who were active in health-related peer-to-peer online communication remained stable [19].

Next to empowerment as an important indicator for how patients cope with their disease, the psychological processes of patients is also of importance. From expressive writing literature, it is known that when individuals go through a traumatic experience (such as being diagnosed with cancer), it is important to process this difficult experience in a *healthy* psychological manner [20]. A methodology for investigating these psychological processes through language use is the *Linguistic Inquiry and Word Count* (LIWC). The LIWC has been used in previous work to distill psychological processes from the content of online support communities, by Owen et al [21] and Lieberman [22]. Owen et al used the LIWC to analyze the content in an online coping skills training group for women with breast cancer and related the LIWC analysis to questionnaires about well-being. They found that the use of words related to cognitive processes (ie, uncertainty and logic) and affective processes (ie, anxiety, sadness, anger, and positive emotions) was significantly associated with improved emotional well-being. Lieberman analyzed the relation of 1 specific LIWC category, *insightful disclosure* (a subcategory of cognitive processes based on 116 words such as *aware*, *know*, and *realize*) to 4 outcome dimensions: depression, functional well-being, physical well-being, and breast cancer concerns. They found that for all the 4 outcome measures, insightful disclosure played a role.

In this study, we investigated the representation of LIWC categories in forum posts and the presence of empowerment processes to establish the relationship between empowerment and the psychological processes expressed. In other words, we investigated to which extent empowerment processes are co-occurring with textual indicators for psychological processes. We used the Dutch version of the LIWC, which was developed by Zijlstra et al [23].

### Goals and Research Questions

The goal of this study was to quantify the presence of empowerment processes in the online forum discussions by people affected by cancer, using automated text analysis techniques.

We address the following research questions:

1. To what extent is the intensity of online participation correlated to indicators of empowerment processes in user-generated content on an online cancer patient discussion forum?
2. Are different aspects of empowerment related to different types of psychological processes, indicated by word use?
3. What are the differences in frequencies of empowerment patterns for patients with different forms of cancer?

## Methods

### Data Collection

We obtained a sample of the discussion forum of the Dutch online community Kanker.nl. Kanker.nl is an initiative of the Dutch Cancer Society, the Netherlands Comprehensive Cancer Organisation, and the Dutch Federation of Cancer Patient Organizations. These 3 major cancer organizations have joined forces in 2012 to provide a single platform where people who have or have had cancer and their loved ones can find reliable medical and health information and exchange experiential knowledge about cancer. The discussion forum is 1 of the 3 main pillars of kanker.nl, together with a library and a collection of blogs. The forum sample consisted of all published posts at the kanker.nl discussion forum up until the start of this research project (November 23, 2016).

### Data Coding and Annotation

We labeled the posts in our sample with 2 types of labels: labels referring to empowerment processes and labels denoting LIWC categories. Both groups of labels were automatically assigned to the posts. Automatic labeling of the empowerment processes was done by text classifiers trained on a manually labeled subsample. For the automatic labeling of the LIWC categories, we used the Dutch version of the LIWC consisting of a total of 66 word categories that are assigned to text based on occurrences of words in the text. Both forms of labeling are described below in more detail. After the automatic labeling with both types of labels, we investigated (1) the relationship between empowerment processes and the intensity of online participation, (2) the relationship between empowerment processes and the LIWC categories, and (3) the differences between patients with different types of cancer.

### Labeling Forum Posts With Empowerment Processes

#### Selecting Empowerment Constructs for Manual Coding

To answer our research questions, we needed a forum sample with annotated empowerment processes. We developed a coding scheme consisting of the 5 previously listed empowerment processes derived from the literature that are relevant in the present context. We decided to define external source as a separate category besides informational support, because a reference to an external source can also be posted independent of a question, for example, when a user points to an interesting publication in the media. Posts containing these references often do not provide information in the post texts. Thus, we included in our study the following 5 empowerment constructs that occur in our forum sample: narrative, question, informational support, emotional support, and external source. In the remainder of this paper, we refer to these 5 categories as empowerment constructs. We first created a sample of manually labeled posts with this coding scheme. Using the labeled data, we then trained and evaluated classifiers with which we automatically labeled all posts in the corpus. This allowed us to quantitatively analyze empowerment constructs in the forum on a large scale.

This process is discussed step by step in the next 4 subsections.

##### Manual Annotation

We randomly selected 2051 forum posts from the Kanker.nl data to be manually annotated. From these 2051, 114 were coded by 2 raters to compute the reliability of the data in terms of interrater agreement. We used the Radboud Research participation system to recruit students as raters and additionally hired 5 paid student assistants. We created an online tool to annotate the data [24]. In our annotation scheme, 1 post can have multiple empowerment constructs; thus, the posts are annotated with respect to each of the empowerment constructs as present in the post (*yes*) or not (*no*). The annotators were allowed to leave the answer to a question undecided (select neither *yes* nor *no*) if they were unsure about the presence of the empowerment construct.

##### Classifier Learning

As a post can be labeled with more than 1 empowerment construct, we trained 1 binary classifier per empowerment construct, with the labels being *yes* (construct is present in the post) and *no* (construct is not present in the post). As features, we used all words from the post, after we lowercased the text and removed punctuation. One exception is that we replaced the ? by the token question_mark. We did not remove stop words. Stop words are highly frequent words, typically function words (eg, as, of, with, and the), which are commonly removed for text categorization into topical categories because they bear little content. We do not remove stop words because we expect function words such as pronouns to play a role in the expression of empowerment constructs.

We ignored all the empty fields (the annotator chose neither yes nor no), which cause the number of example items to differ per construct. To avoid overfitting, we split the data into 2 partitions: 80% for training the classifiers and 20% for evaluating them. Thus, for each construct, we split the data in a training set (80% of the examples) and a held-out test set (the remaining 20%). From the 114 items that were labeled by 2 annotators, we included in the training set only the items where the raters agreed to avoid having conflicting training data. In the test set, we did include the items where the raters did not agree (value for 1 of the 2 raters), because the quality of the classifier would be overestimated if only the agreed (clear) instances were included.

We used scikit-learn in Python to train and validate the classifiers, 1 for every empowerment construct [25]. We experimented with 6 different classification methods and decided on the use of linear support vector classification (SVC) [26] because it gave the best classification results in terms of precision, recall, and F1, which is the harmonic mean of precision and recall.

Linear SVC has 1 hyperparameter (c). We used 25% of the training set for optimizing c, training on 75% of the train set, and evaluating different values of c on the remaining 25%. We experimented with a grid ranging from c=10^−3^ to c=10^3^ in steps of ×10, as suggested in the documentation of scikit-learn [27]. We found c=1.0 to be the optimal value in terms of F1-score (averaged over the 5 binary classifiers for the empowerment constructs); thus, we used c=1.0 when training linear SVC on the full training set (80% of all labeled data), evaluating on the held-out test set (20% of all labeled data).

##### Automatically Labeling the Corpus With Empowerment Constructs

Provided that the precision of the classifiers was sufficient (>80%), we trained SVC classifiers on all manually labeled dataset and applied them to all unlabeled posts in the corpus. SVC has a natural cutoff for assigning a label in binary classification: if the predicted value is larger than 0, the label *yes* is assigned, and if the predicted value is smaller than 0, the label *no* is assigned. This way, we automatically labeled the complete corpus with empowerment constructs. The 5 classifiers for the empowerment constructs operate independently of each other, meaning that each message is labeled with 0 or more empowerment constructs.

### Labeling Forum Posts With Linguistic Inquiry and Word Count Categories

LIWC analyzes texts for indicators of psychological processes [28]. These indicators are occurrences of words. The LIWC dictionary defines which words are indicators for which linguistic or psychological category. The linguistic LIWC categories are categories such as first-person singular pronouns and past tense verbs. The psychological LIWC categories are categories such as positive emotions, negative emotions, and anxiety. Examples of indicator words are *me* for first-person singular pronouns and *pain* and *fear* for negative emotions. One limitation of this approach is that 1 word can have multiple meanings, depending on its context. For example, the word *well* could occur in positive (*feeling well*) and neutral contexts (*as well as*), and it could even be a noun (a source of water).

We used the Dutch version of the LIWC consisting of a total of 66 word categories that belong to 4 overarching groups of categories: (1) standard linguistic dimensions (eg, personal pronouns, first-person singular pronouns, and past tense verbs), (2) psychological processes (eg, positive emotions and anxiety), (3) relativity (time and space), and (4) personal concerns (eg, work, money, and religion). The categories are organized hierarchically. For example, the main category *cognitive processes* under *psychological processes* has several subcategories, among which *insightful disclosure*, *inclusive*, and *exclusive*. Due to this hierarchy, a word can belong to more than 1 category. For example, the word *ik* (*I*) occurs in the category *pronoun* as well as the category *1st-person singular*.

A forum post can have more than 1 LIWC category assigned to it, based on the words occurring in the post.

### Data Analysis

#### Relating Empowerment Processes to the Intensity of Online Participation (Research Question 1)

We investigated the relationship between each of the empowerment constructs in the automatically labeled forum and the intensity of online participation. The most straightforward metric for intensity of participation is the number of messages that a member has posted. In addition, we also considered the average post length to be of relevance: a user who posted only short messages might be less involved in the community than a user who posts more lengthy messages. We also took into account 2 measures for a user’s social relations in the community: the number of contacts and the number of *incoming* contacts (the number of users who follow this user). The latter is an indication of popularity. Thus, we related empowerment processes to 4 quantitative user activity characteristics: number of posts, average post length, number of contacts, and popularity.

To quantify the relations, we converted the label counts for the empowerment constructs per user to relative label counts, by dividing the number of occurrences of a label for the user by the total number of posts by the user. For example, a user might have 8 posts, with the following relative label counts of the 5 empowerment construct labels: narrative 0.5, question 0.125, informational support 0.0, emotional support 0.75, and external source 0.5.

We then computed the correlation in terms of Kendall τ between the user characteristic (eg, the number of posts) and the relative label count (eg, 0.125 for *question*).

#### Relating Empowerment Processes to Linguistic Inquiry and Word Count Categories (Research Question 2)

Once we completely annotated the corpus with empowerment constructs and with the LIWC categories, we investigated the correlations between the 2 types of variables. To that end, we created a table with for each post (N=5532) 5 columns. Each column denotes the presence (1 or 0) of each of the empowerment constructs (narrative, question, informational support, emotional support, and external source) according to the automatic classifiers and 20 columns for the relative frequencies of the 20 most frequent LIWC categories. The relative frequency of a LIWC category for a post is defined as the numbers of occurrences of all words from the category in the post divided by the total number of words in the post.

We then performed 5 separate logistic regression analyses (in *R*), 1 for each empowerment construct. Thus, in each analysis, the presence of an empowerment construct (true or false) is the dependent variable and the 20 LIWC categories are the independent variables. In this way, we can investigate which LIWC categories contribute to which empowerment variables. From the resulting regression models, we removed all variables with negative coefficients and all variables that are not significantly contributing to the model (*P*>.01).

#### Differences in Empowerment Patterns for Different Types of Cancer (Research Question 3)

Previous research has suggested that patients with different types of diseases have different online social support needs [29]. We investigated the differences in empowerment processes for patients with different cancer types by investigating the occurrences of empowerment processes for the 5 most occurring cancer types in our forum sample: breast cancer, lung cancer, colorectal cancer, gynecological cancer, and prostate cancer.

## Results

### Collected Sample

The collected sample comprises 5534 posts in 1708 threads by 2071 unique users, posted between April 17, 2013, and November 23, 2016. The threads are organized in 38 categories. The forum does not focus on 1 particular cancer type; over 15 cancer types are represented, the largest being breast cancer (760 posts), lung cancer (423 posts), and colorectal cancer (389 posts). In total, 1356 authors only posted 1 post and 33 posted over 20 posts. In the sample, user names were replaced by unique keys. There was no identifying information of the forum users available to the researchers during the analyses.

### Data Quality

#### Interrater Agreement

We report interrater agreement for the subsample that was annotated by 2 raters. The absolute agreement is defined as the number of items for which both raters agree divided by the number of items for which both raters selected a value (yes or no). Cohen kappa weighs the absolute agreement with the *chance agreement* based on the number of *yes* and *no* values for the empowerment constructs. For data that have a strong class imbalance, Cohen kappa is low, because the chance agreement is high (if both raters almost always select *no*, then there is a high chance that they both assigned *no* for a given item). A kappa value higher than 0.4 indicates moderate agreement; a kappa value higher than 0.6 indicates substantial agreement.

Table 1 shows the results for the empowerment constructs. The table shows that the interrater agreement is the lowest for informational support. This might be because this construct has the least explicit textual indicators. For the other 4 constructs, the agreement is substantial (Cohen kappa >0.6).

#### Classifier Evaluation

We report precision and recall for the *yes* categories for each construct as evaluation measures:

- Given construct X, *precision* is the percentage of posts automatically labeled with X=yes that also have the label X=yes in the human-labeled data (true positives/[true positives+false positives]). Precision gives the proportion of the automatically assigned labels that are correct.
- Given construct X, *recall* is the percentage of posts with the label X=yes in the human- labeled data that were also automatically been labeled with X=yes (true positives/[true positives+false negatives]). Recall gives the proportion of true labels has been found automatically.

The results are provided in Table 2. The overall results are good. The average precision over constructs is 85.6%, which means that of the 100 assigned labels, 14 are incorrect (averaged over the constructs). The results also show that some constructs are easier to classify than others, but precision scores are all between 75% and 93%. The recall scores are lower (except for narrative); informational support and external source are missed quite often by the classifiers. Considering the goal of the automatic labeling (analysis of the labeled corpus), we consider precision to be more important than recall—it is more problematic to assign wrong labels than to miss labels because wrongly assigned labels might lead to unjustified conclusions. Moreover, the classifiers trained on all labeled data (instead of the 80% training set) are likely to be a bit better because they have more examples available. Therefore, we consider the quality of the classifiers sufficient for labeling the complete corpus.

**Table 1:** Interrater agreement results for the empowerment constructs

Empowerment construct	Number of items^a^	Measured agreement, %	Cohen kappa
Narrative	112	0.86	0.71
Question	58	0.90	0.79
Informational support	65	0.72	0.40
Emotional support	57	0.93	0.65
External source	65	0.86	0.68

^a^Recall that the number of example items differs per construct because we ignore all the empty fields (the annotator chose neither yes nor no).

**Table 2:** Overall evaluation of the classifiers for the empowerment constructs, in terms of precision, recall, and F1 (the harmonic mean of precision and recall).

Empowerment construct	Precision, %	Recall, %	F1, %
Narrative	89.2	93.2	91.1
Question	87.2	62.4	72.7
Informational support	75.0	52.0	61.4
Emotional support	83.6	65.7	73.6
External source	93.0	55.9	69.8
Average over constructs	85.6	65.8	73.7

#### Statistics of the Automatically Labeled Corpus

On average, messages in the corpus were assigned 1.4 labels. Table 3 shows the distribution of empowerment constructs in the automatically labeled corpus.

An example message text for each empowerment construct is listed below:

- Narrative: “My husband has invasive bladder cancer not operable. Now has a urine stoma that was OK to live with. But recently he got 2 kidney drains that constantly leak.”
- Question: “How are you feeling about your scar after the operation? Are you embarrassed or do not care? I’m curious about your comments.”
- Informational support: “After radiotherapy in the head and neck area there is a good chance that the salivary glands are also blasted, giving you a drier mouth and also a different chemical composition of the saliva.”
- Emotional support: “What a horribly scary time your mother (and all of you) is going through! Terrible to always be in suspense whether or not the chemotherapy has done its work, very recognizable!”
- External source: “I saw this movie from SchoolTV via NLNet (patient association for people with lymphedema). It gives a clear explanation about lymphedema. Useful for patients themselves, or to show others if you find it difficult to explain (or do not feel like it ;-)).”

### Results for Research Question 1, Empowerment, and Intensity of Online Activity

Table 4 shows the correlations in terms of Kendall τ between the user characteristics and the relative label count for each of the empowerment constructs. The correlations that are not significant (*P*>.05) are not shown.

The correlations indicate the number of posts is the strongest indicator of the empowerment constructs: users with more posts more often refer to an external source and provide informational support and emotional support (all correlations above 0.2) and less often share narratives (negative correlation). The relation with asking questions is weak (below 0.1).

**Table 3:** Distribution of assigned empowerment constructs in the automatically labeled corpus (N=5532). Note that the percentages do not sum to 100% because a post can have more than 1 label assigned to it.

Empowerment construct	Frequency of posts, n (%)
Narrative	3482 (62.94%)
Question	1318 (23.83%)
Informational support	1521 (27.49%)
Emotional support	855 (15.46%)
External source	753 (13.61%)

**Table 4:** The significant correlations (in terms of Kendall τ) between the frequency of an empowerment construct for a user and 4 user variables. In all cases, N=2071 (number of users who posted at least one message). Correlations with *P*>.05 are not shown. *P* values are shown for correlations with a significance of .001<*P*<.05.

Empowerment construct	Correlation with user variables, Kendall τ			
	Number of posts	Average post length	Number of contacts	Popularity
Narrative	−0.297^a^	0.232^a^	−0.126^a^	−0.117^a^
Question	0.065^a^	−0.105^a^	—^b^	—
Informational support	0.204^a^	0.072^a^	0.086^a^	0.090^a^
Emotional support	0.232^a^	0.037^c^	0.168^a^	0.168^a^
External source	0.255^a^	—	0.149^a^	0.160^a^

^a^*P*<.001.

^b^Correlations with *P*>.05 are not shown.

^c^*P*=.03

### Results for Research Question 2, Empowerment, and Linguistic Patterns

Table 5 shows the results from the logistic regression analyses, predicting the presence of an empowerment construct from the relative frequencies of the 20 most frequent LIWC categories. The Dutch LIWC categories and the example words from Zijlstra et al [23] were translated here for the reader’s convenience.

The table shows that a number of LIWC categories have significant correlations with the empowerment constructs. Not all correlations are interesting and easy to interpret. For example, questions contain many pronouns, and informational support is correlated with expression of leisure. Others are more interesting: narratives contain especially first-person singular and third-person pronouns and also correlate with the expression of religion. Emotional support contains more second-person references and words expressing assent (eg, *ok* and *yes*) and emotional processes (expressions of feelings). External sources also contain more second-person references and correlate with cognitive processes (eg, knowing and thinking).

### Results for Research Question 3: Differences Between Patients With Different Cancer Types

**Figure 1:**
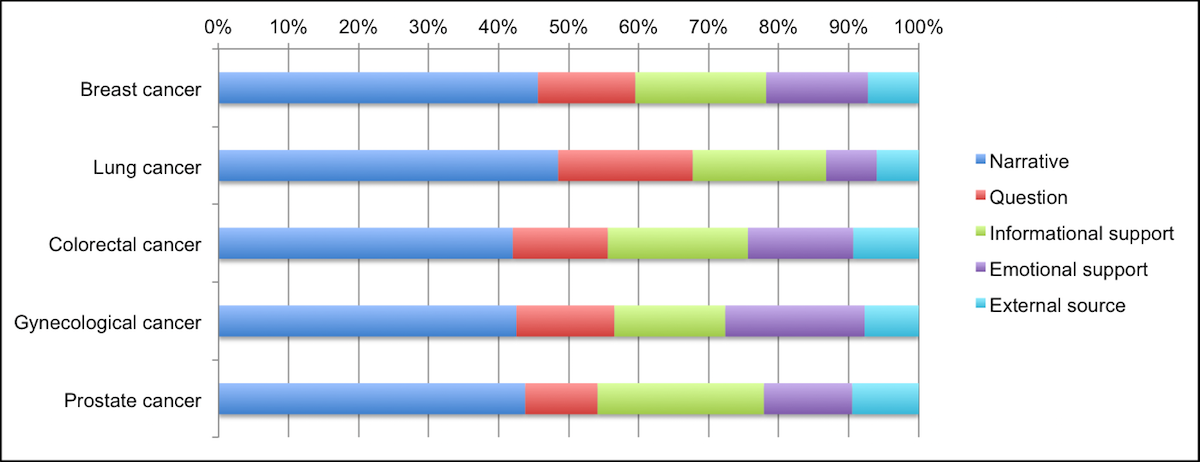
Distribution of occurrences of empowerment processes in the automatically labelled corpus, for the 5 most occurring cancer types.

We investigated the differences between patients with different cancer types in our data by separately counting the occurrences of empowerment processes for the 5 most occurring cancer types in our forum sample: breast cancer, lung cancer, colorectal cancer, gynecological cancer, and prostate cancer. The resulting distributions are shown in Figure 1. The figure shows that although most relative frequencies are similar between the cancer types, patients with lung cancer ask more questions and provide less emotional support than patients with other common cancer types.

**Table 5:** Estimated regression coefficients for the Linguistic Inquiry and Word Count categories that are significant (*P*<.01) positive predictors for predicting the presence of an empowerment construct. *P* values are shown for predictors with a significance of .001; *P*<.01.

Linguistic Inquiry and Word Count category and subcategory (with 3 example words per subcategory)		Estimated regression coefficients				
		Narrative	Question	Informational support	Emotional support	External source
**I Standard linguistic dimensions**						
	Total pronouns (I, you, our)	—^a^	3.11^b^	—	—	—
	1st person singular (I, me, my)	5.43^b^	—	—	—	—
	Total 2nd person (you, your)	—	—	3.16^b^	4.15^b^	4.67^b^
	Total 3rd person (she, he, them)	4.94^b^	—	2.19^b^	—	—
	Negations (no, never, not)	1.93^b^	—	1.13^c^	—	—
	Assent (yes, OK)	—	—	—	2.04^c^	—
**II Psychological processes**						
	Emotional processes (happy, sad, miserable)	—	—	—	1.35^b^	—
	Cognitive processes (know, cause, think)	—	—	—	—	1.27^b^
	Senses and perceptual processes (see, feel, hear)	—	0.85^b^	—	—	—
	Social processes (communicate, share, help)	1.69^b^	0.45^c^	—	—	—
**Relativity**						
	Time (summer, previously, as soon as)	0.79^c^	—	—	—	—
	Space (close, place, north)	—	—	—	—	1.00^d^
**Personal affairs**						
	Leisure (cycling, fitness, training)	—	—	3.90^b^	—	—
	Religion (baptism, prayer, catholic)	2.77^b^	1.09^b^	—	—	—

^a^Not applicable, as *P* values are only shown for predictors with a significance of .001<*P*<.01.

^b^*P*<.001.

^c^*P*=.002.

^d^*P*=.009.

## Discussion

### Principal Findings

In this paper, we presented methods to analyze empowerment processes in an online discussion forum in a structured, largely automatic way. We implemented and evaluated 2 automated methods for analyzing the content of an online cancer patient community: (1) word-based text classifiers for coding forum posts with empowerment constructs, using a manually coded subsample as training data, and (2) LIWC, an unsupervised (dictionary-based) analysis technique that was designed to distill psychological processes about user-generated content.

This paper shows that the theoretical construct patient empowerment can be operationalized and measured in online communication using automatic classifiers trained on a sample of manually labeled data. This implies that other theoretical constructs (in health care) on the patient level, such as health literacy or experienced quality of care, and care concepts related to governance, such as integrated care or access to health care, might be studied on online discussion forums if researchers are interested in the patients’ perspective. When these types of analyses become further refined, they can be a cost-efficient way for policy makers to take account of the issues that are relevant in respective patient groups when setting the agenda for change or initiating improvements in health care.

In the remainder of this section, we answer our research questions.

### Research Question 1. To What Extent Is the Intensity of Online Participation Correlated to Indicators of Empowerment From User-Generated Content on an Online Cancer Patient Discussion Forum?

We succeeded in distilling different types of empowerment processes from a peer-to-peer cancer patient forum. It was possible to automatically annotate a corpus of over 5500 messages on the message-level, by training a classifier on a smaller sample of manually created example data.

We observed empowerment constructs in the online conversations, and based on the linguistic associations, we conclude that online peer to peer contact fulfills the need for personal contact with others in a similar situation. Overall, sharing of personal stories with peers (narrative) was the most frequently observed process. Other empowerment processes we studied were providing peers with informational or emotional support, answering their questions, or referring them to external sources. Users that are more active online in terms of number of posts and number of contacts more often guide other users to external sources of information and provide more support than less active users.

### Research Question 2. Are Different Aspects of Empowerment Related to Different Types of Psychological Processes, Indicated by Linguistic Patterns?

The combination of LIWC with the empowerment constructs has yielded a number of new insights. We found that the narrative is an important empowerment construct and that this is a means for participants to relate to each other and the context. Being ill requires redefining of one’s position to the rest of the world and finding a way to deal with this new situation [11]. In this study, this appears to take the form of talking about the relationships that the patients have with others around them. In terms of linguistic constructs, we observed that personal pronouns are related to empowerment constructs, indicating that online empowerment processes strongly fill a need to relate the personal situation to the context. The narrative is related to both the first-person and third-person pronoun, indicating that sharing a narrative is a means to share personal experience and to link this experience to others. It could be indicating that sharing a personal story is a means to reach out to others.

### Research Question 3. What Are the Differences in Frequencies of Empowerment Patterns for Patients With Different Types of Cancer?

We found no striking differences between the frequencies of empowerment patterns for patients with different types of cancer: Most relative frequencies are similar between the cancer types; the only category that is slightly different from the others is the group of patients with lung cancer. They ask more questions and provide less emotional support than patients with other common cancer types. One aspect that might play a role here is that of these cancer types, lung cancer has the worst prognosis: 30% of the patients diagnosed with lung cancer are still alive 3 years after the diagnosis, as opposed to 70%-95% for the other cancer types.

### Comparison With Prior Work

Most of the previous studies concerning online empowerment and online social support use qualitative methods to study online content [6,14-17] or established methods such as questionnaires [3,18,19]. These studies provide knowledge on empowerment processes, the underlying mechanisms, and the empowerment outcomes. The dominant role of narratives as empowerment process in patient support groups has been found in previous studies as well [30]. Previous text mining studies show that it is possible to identify (disease-related) topics that are discussed online. Birnbaum et al [28] identified self-report of schizophrenia from Twitter messages. Nzali et al [21] compared results from text mining techniques applied on social media with results from self-administered questionnaires and found good correspondence between detected topics on social media and topics in the questionnaires.

Our unique methodological contributions compared with previous studies are twofold: (1) we are the first to successfully apply text classification to the task of labeling forum posts with empowerment constructs and (2) we show the correlations between LIWC categories and empowerment processes in forum posts.

The combination of LIWC with the empowerment processes confirmed a number of findings from previous works. We found, for instance, that the narrative is an important empowerment process. Being ill requires redefining of one’s position to the rest of the world and finding a way to deal with this new situation [12]. In this study, this process appears in the form of talking about the relationships that the patients have with others around them, as the LIWC categories indicating relationships are prominently present in our analyses.

With respect to the development of user activity over time, Wang et al [18] showed that the participation rate in online communities dropped steeply in a short time span after a user’s registration and that most participation was related to the narrative of the user’s own situation. We found similar patterns in the relationship between empowerment processes and user activity, suggesting that new members of a community mainly share their own stories, whereas more experienced and active members provide social or informational support more frequently. This finding is in line with previous research. Coulson [17] found that older and more active users often take a more senior role in which they respond to questions of new users and thereby provide hope and encouragement (ie, emotional support). As Lasker et al [31] puts it: “posts from more ‘senior’ peer experts [long-active members] may provide role models for newer members”. On the topic of narratives, Wang et al [32] showed the important role of narratives, the sharing of ones’ status in online communities. They found that narratives can be used to both elicit (emotional) responses by using the narratives as a thread opening as well as a way to respond to questions from other users.

These findings suggest that persistent and active support group participation might contribute to experiential and informational empowerment, a conclusion that fits with the findings from a study on the relationship between online support group participation and emotional well-being over time [33]. Results from that study showed that being active online might especially benefit patients who do not actively approach their emotions naturally, suggesting that peer-to-peer forums might *teach* patients how to deal with illness.

### Limitations

The findings presented in this paper are subject to some limitations. The forum posts that we included contained for obvious reasons only utterances from patients that are present online. The estimations of percentages of active and nonactive online group members differ from 1% to 10% [34], to a quarter active users [5], to about half of the group members [35,36]. It has been found that posters report higher levels of empowerment than lurkers, even though lurkers also benefit from reading the forum texts [5,35,36].

This study used data from a general cancer patient forum, and therefore, we involve a more diverse user group than previous studies addressing groups of patients having 1 type of cancer (eg, breast cancer [21]). On the other hand, our study was limited to patients who actively participate in an online discussion forum. These patients are usually younger and higher educated than the average of the population [37]. This might imply that we studied the group of patients who are more empowered, more actively seeking online information, and more actively interacting with peers.

Empowerment is a much-used term, with many different definitions [38,39]. We limit our study to the analysis of empowerment *processes*, as they are likely to take place on the internet, whereas the empowerment *outcomes* will take place in interaction with physicians and insurers. Previous research found that patients experience both processes and outcomes, and this might indicate that both are related [12,40]. We found that most often forum users relate to their personal story, to exchange personal experiences and relate emotionally and socially to one another. References to external sources also occur frequently. This means that the information aspects of empowerment also take place in other parts of the internet apart from the forum itself.

In addition to that, this study focused on patients with cancer. Even though we assumed that the empowerment processes are similar between people who experience life-threatening diseases, more research needs to confirm whether the results in this study are generalizable to patients with other diseases.

We also acknowledge limitations of the methods that we applied for the analysis of the forum sample: the limitation of text classifiers is that they need training data (manual coding)—the more training data, the better the quality of the classifier. Hence, text classification is not a method that can be applied without any supervision. A known limitation of the LIWC is that it is based on word occurrences. This means that it does not take combinations and contexts of words into account, which are particularly relevant for negations (ie, *not*) and ambiguous words (ie, *well*).

### Conclusions

In this paper, we studied empowerment processes in online peer-to-peer communication and showed that different empowerment processes are associated with intensity of online use. The combination of linguistic analyses with measurement of empowerment provided indications that online patient empowerment helps users to relate to peers and redefine their situation in addition to giving informational and emotional support.

We recommend the further use of text mining in future work addressing the online activities of patients, because it enables the analysis of large amounts of unsolicited data. Our study showed that quantitative content analysis can give interesting insights, with respect to empowerment, language use, and psychological processes.

## Abbreviations

LIWC: Linguistic Inquiry and Word Count
SVC: support vector classification
